# Changing gender roles and attitudes and their implications for well-being around the new millennium

**DOI:** 10.1007/s00127-013-0730-y

**Published:** 2013-08-02

**Authors:** Helen Sweeting, Abita Bhaskar, Michaela Benzeval, Frank Popham, Kate Hunt

**Affiliations:** 1 MRC/CSO Social and Public Health Sciences Unit, University of Glasgow, 4 Lilybank Gardens, Glasgow, G12 8RZ UK; 2 Institute for Social and Economic Research, University of Essex, Colchester, CO4 3SQ UK

**Keywords:** Gender roles, Attitudes, Well-being, Gender differences, Age and period effects

## Abstract

**Purpose:**

Given evidence that gender role attitudes (GRAs) and actual gender roles impact on well-being, we examine associations between GRAs, three roles (marital status, household chore division, couple employment) and psychological distress in working-age men and women. We investigate time-trends reflecting broader social and economic changes, by focusing on three age groups at two dates.

**Methods:**

We used British Household Panel Survey data from 20- to 64-year-olds in heterosexual couple households in 1991 (*N* = 5,302) and 2007 (*N* = 6,621). We examined: levels of traditional GRAs according to gender, age, date, household and employment roles; associations which GRAs and roles had with psychological distress (measured via the GHQ-12); whether psychological distress increased when GRAs conflicted with actual roles; and whether any of these associations differed according to gender, age or date.

**Results:**

Gender traditionalism was lower among women, younger people, those participating in 2007 and in ‘less traditional’ relationships and households. Psychological distress was higher among those with more traditional GRAs and, particularly among men, for those not employed, and there was some evidence of different patterns of association according to age-group. There was limited evidence, among women only, of increased psychological distress when GRAs and actual roles conflicted and/or reductions when GRAs and roles agreed, particularly in respect of household chores and paid employment.

**Conclusions:**

Although some aspects of gender roles and attitudes (traditionalism and paid employment) are associated with well-being, others (marital status and household chores), and attitude-role consistency, may have little impact on the well-being of contemporary UK adults.

**Electronic supplementary material:**

The online version of this article (doi:10.1007/s00127-013-0730-y) contains supplementary material, which is available to authorized users.

## Introduction

Over the latter part of the twentieth century and into the first decades of the twenty-first century, societal gender role attitudes (henceforth GRAs, also termed gender role beliefs or ideology) have become more egalitarian among both men and women [1], paralleling broader social and economic changes. There have been striking increases in the proportion of adults choosing to cohabit rather than marry [2] and also, among women, particularly those with children, in the proportion in employment (UK employment rates in 1974 and 2003, respectively, were 95 and 86 % in men, 67 and 73 % in childless women and 36 and 58 % in mothers) [3]. In contrast, although men’s involvement in domestic work rose from the 1960s, it reached a plateau in the mid 1990s, changing little in the following decade [1].

The implications of these changes in attitudes and roles for other aspects of life are not well understood. In particular, it has been suggested that ‘internalisation of sex roles and gender stereotypes and the ramifications of these roles, both of which can be measured at an individual level, are rarely among the inputs studied when health is the output’ (p. 370) [4]. Changes in GRAs and roles, or changes in the meanings associated with particular roles are, therefore, important in respect of the impact they might have on patterns of psychological distress in men and women [5, 6]. In this paper we focus on how GRAs and indicators of men’s and women’s actual roles in the home and the labour market are associated with psychological distress. Inclusion of both GRAs and roles means we can investigate the relative importance of each. Analyses are based on data from the UK British Household Panel Survey (BHPS) which allows us to look at men and women from three different working age groups (20–34, 35–49 and 50–64) at two different dates (1991 and 2007).

### Gender roles and attitudes: patterning and associations with well-being

Traditional GRAs privilege men’s roles in paid work and their status as the family ‘breadwinner’, while assuming women should prioritise caring for the home and family over other roles. Egalitarian GRAs, in contrast, support equality in all domains [7]. More traditional GRAs are more common among men [7–9] and older generations [10–12]. Several studies suggest they may be also associated with greater psychological distress. For example, more traditional GRAs were associated with poorer well-being among ‘Dutch mainstreamers’ and both Caribbean and Mediterranean immigrant men and women living in the Netherlands [8], while a study of 45- to 79-year-olds in the UK found GRAs was unrelated to mental health among men, but women with more traditional GRAs had poorer mental health [13]. Another UK study found more traditional GRAs were positively associated with suicidal thoughts in early and late middle-aged cohorts [14].

Existing evidence on gender-related roles rather than attitudes is very mixed. Shared household responsibilities are more likely among those with more egalitarian beliefs and higher levels of education, and among childless couples where both partners are working [8, 15–17], although there is some evidence from Sweden that the association between parenthood and traditional gender differences in household tasks might be changing [18]. Several studies have reported lower well-being among both men and women who spend more time on housework, who live in households where household responsibilities are shared less equally and/or who perceive that household responsibilities are not equally shared [8, 19, 20]. However, some find no associations between the actual division of household labour and well-being [21]. Others find that poorer mental health among both men and women is associated with only certain types of domestic work such as routine and unavoidable (‘female’) tasks, but not with tasks such as gardening or home repairs which are more commonly undertaken by men [5]. Still others have suggested that well-being is related to other forms of ‘family work’ (such as childcare or ‘emotion work’ like suggesting solutions to their partner’s problems), but not housework [22]. Some studies have found associations between measures of actual or perceived levels of housework and marital satisfaction or well-being among women but not men [19, 23–26]. The role of paid employment, which among women is more likely among those with more egalitarian GRAs [27], is generally associated with lower psychological distress among both men and women [19, 28–30].

However, it is plausible that roles and attitudes should be considered in tandem, in respect of their relationships with well-being. In particular, consistency between attitudes and roles (i.e. whether an individual’s GRAs as more traditional or egalitarian are in line with their household and paid employment roles) may be important for predicting well-being. This notion can be traced back to the observation by Komarovsky [31] during the 1930s that unemployed American men were more likely to suffer depression if they had a traditional economic provider and ‘boss’ self-identity than if they perceived their role as father and husband was more important. All but two [1, 25] of the studies of this ‘fit between self and situation’ (p. 638) [32].which we have identified have been conducted in the US and many have been based on small samples. Most focus on ‘fit’ between GRAs and household chores with marital satisfaction as the ‘outcome’, and a smaller number examine GRAs and employment status. Surprisingly, none have investigated another role which might plausibly be linked with GRAs in association with well-being, namely marital status.

Most studies of attitude-role inconsistency find that attitudes have a moderating effect on the relationship between employment and/or household chores and well-being, although there are a few exceptions [5, 25, 33]. We are aware of only one UK study in this area, based on analysis of data from participants in the 2002 and 2006 British Social Attitudes Surveys who were married/cohabiting and employed. It found that women categorised as ‘incongruent liberal’ (with egalitarian GRAs but more traditional division of household chores) were more likely to report disagreement over chores, while ‘congruent liberals’ (egalitarian GRAs and more egalitarian division of chores) were more likely to report a lack of stress at home. No such associations were found among men [1]. Several other (US) studies also suggest that inconsistency between GRAs and household chore division is associated with poorer well-being; most such studies have focused on women. For example, unequal division of housework was related to lower perceived spousal support and lower psychological well-being among egalitarian but not traditional wives [34]. Unequal housework division was also associated with perceived unfairness and poorer reported marital relationships, again in egalitarian but not traditional wives [35]. Another study found receipt of practical support in the home from a husband was associated with self-assessed marital quality more strongly among egalitarian than traditional wives [7], while among traditional, but not egalitarian wives, those whose husbands did more child-care than they had expected prenatally had higher levels of psychological distress [21]. A study of husbands found those with more traditional beliefs who performed fewer chores and those with more egalitarian beliefs who performed more chores had higher marital satisfaction than those whose beliefs and roles conflicted [17]. Finally, among members of couples with new babies or young children, marital satisfaction was lower for those with more traditional attitudes but more egalitarian division of household chores [16].

Among the smaller number of studies focusing on GRAs and employment status, analyses have also found conflicting attitudes and roles to be associated with psychological distress. Thus, a study which measured symptoms of depression found wives were less depressed when their preferences for doing paid work or not were consistent with their actual employment status and husbands were less depressed when their wives’ employment status matched what they stated they would prefer their wives to be doing [36]. Similarly, among working wives, ‘ambivalent co-providers’ (who realised their income was necessary but believed their husband should be the main breadwinner) had lower levels of marital satisfaction than those who believed in shared financial responsibility [37]. Among women with more egalitarian views, psychological distress was greater among housewives compared with those in employment [32] and those who returned to work part-time rather than full-time after childbirth [21].

Secular changes add further complexity and, as noted earlier, there is evidence of substantial differences in the experiences of people from different generations, even those not far apart in age. Thus, in the UK, there have been major changes in patterns of marriage and cohabitation, family formation, education and female employment since the mid Twentieth century [11, 38]. However, studies of GRAs, roles and well-being have not paid attention to generational differences, nor whether having views which conflict with prevailing cultural trends and expectations is important. As gender relations and gender roles have changed over time, we might expect generational differences in the associations which GRAs and what we have termed ‘couple roles’ (specifically marital status, the gender balance of household chore performance and of the couple’s employment) have with psychological distress. For example, it has been suggested that those less committed to a particular identity will be less psychologically distressed by household arrangements which conflict with that identity [15], and it may be that for younger generations of women, egalitarian GRAs are so taken for granted [39] that they are actually less important. Consistent with this, one study found that education and employment status were strong predictors of GRAs in two older cohorts of women (aged 63–71 and 42–50 in 1996), but not in a younger cohort (aged 18–26) [40], and another that GRAs were associated with suicidal thoughts in early and late middle-aged cohorts, but, again, not in a younger cohort [14].

### This paper

Our paper is based on data from the BHPS, as are several other studies in this area [10, 13, 27, 41]. The most recent analysis (2011) and by far the most relevant here, examined how gender, family-related variables and GRAs were associated with psychological distress. The analysis focused on married couples aged 45–79 years who provided data in 2001. It found significantly increased levels of psychological distress among husbands reporting early fatherhood and co-residence with a child/children aged 16 or more, and among wives with traditional GRAs, while having had a child when aged 35 or more reduced levels of psychological distress among wives [13]. Our analysis, which draws on BHPS data obtained in 1991 and 2007, builds on this, using the same measure of psychological distress (the GHQ-12). It includes GRAs and several aspects of ‘couple roles’ (marital status, and the gender balance of both household chore performance and the couple’s paid employment) allowing us to investigate the association of each with psychological distress and, importantly, examine the effects of conflict between GRAs and each ‘couple role’. We include education in our analyses, given its known association with GRAs, ‘couple roles’ [8] and psychological distress [42] and also adjust for the presence of dependent children in the household as this is likely to affect the level and type of household chores.

On the basis of the existing literature we set out to test a number of hypotheses. We expected the following results:

1. Less traditional GRAs in women, younger people and people participating in the more recent BHPS wave [7–12].
2. Less traditional GRAs among people with more education and among those in ‘less traditional’ heterosexual couple relationships (i.e. cohabiting rather than married; the man doing/substantially sharing household chores; the woman employed and/or the man not employed; no dependent children) [8, 15–17].
3. Greater psychological distress among the following groups: those with more traditional GRAs [8, 13, 14]; those reporting the gender-balance of household chores to be less equitable [8, 19, 20] (especially for women [19, 23–26]); and those not in employment [19, 28–30].
4. Greater psychological distress when GRAs conflict with actual roles (i.e. traditional GRAs combined with cohabitation rather than marriage, the man doing more chores and/or the woman as sole breadwinner, or egalitarian GRAs combined with marriage, the woman doing more chores and/or the man as sole breadwinner), again, particularly among women [1, 7, 16, 17, 21, 32, 34–37].

We include data from three different working-age groups (20–34, 35–49 and 50–64) collected at two different dates 16 years apart (1991 and 2007), allowing us to explore whether relationships differ by age and over time.

## Methods

### Sample

Data were taken from Waves 1 (1991) and 17 (2007—the most recent to include items measuring GRAs) of the BHPS, an annual survey of a nationally representative UK sample. The original sample included each adult (age 16+) member of more than 5,000 households, comprising around 10,000 individual interviews. Original sample members have been followed over successive waves; if they move out of their original household, all adult members of their new household are interviewed as are any adults moving in with an original sample member. Booster samples were added for Scotland and Wales in 1999 and for Northern Ireland in 2001. These respondents have been followed up over time and are included in the 2007 sample studied here to maximise our sample size, provided they meet the other eligibility criteria. The survey conforms with the Ethical guidelines of the Social Research Association in respect of confidentiality and informed consent [43].

Since our focus was on attitudes and gender divisions of labour between people in heterosexual couple households, we removed single parents, students, same-sex couples, etc., and also those living in households comprising more than one couple, where the division of roles was likely to be more complex. We removed other adult household members of couple households for the same reason. We also limited our sample to those of working age (20–64 years) and removed ‘proxy’ respondents (in whose respect GRAs were not measured). These exclusions reduced the initial sample sizes from 10,264 to 5,422 (1991) and from 14,910 to 6,934 (2007) (detailed numbers at each stage of this process available in Supplementary Table 1). Limiting the samples to those with complete cases on all variables resulted in final samples of 5,302 (1991) and 6,621 (2007) (see Table 1). Within these numbers there were 1,760 who participated at both dates: 821 from the 20- to 34-year-old age group in 1991 (of whom 750 were aged 35–49, and 71 were aged 50–64, in 2007) and 939 from the 35- to 49-year-old age group in 1991 (who were all aged 50–64 in 2007). Thus the dataset actually comprises 10,163 respondents, 3,542 (35 %) of whom participated only in 1991, 4,861 (48 %) only in 2007 and 1,760 (17 %) at both dates.

**Table 1:** Numbers (and percentages) of men and women in each age group at both dates with tests for significances of: gender differences for each age group at each date; differences between the three age groups for men and women at each date; and differences between the two dates for men and women in each age group^a^

	1991				2007				Date difference		
	Age 20–34 (born 1957–1971), *N* (%)	Age 35–49 (born 1942–1956), *N* (%)	Age 50–64 (born 1927–1941), *N* (%)	Age difference (*χ* ^2^, sig)	Age 20–34 (born 1973–1987), *N* (%)	Age 35–49 (born 1958–1972), *N* (%)	Age 50–64 (born 1943–1957), *N* (%)	Age difference (*χ* ^2^, sig)	Age 20–34 (*χ* ^2^, sig)	Age 35–49 (*χ* ^2^, sig)	Age 50–64 (*χ* ^2^, sig)
**Overall**											
Men—overall	772	1,032	703		702	1,313	1,008				
Women—overall	987	1,127	681		939	1,512	1,147				
**Marital status**											
Men											
Married	583 (75.5)	963 (93.3)	683 (97.2)	209.2, <0.001	352 (50.1)	1,090 (83.0)	921 (91.4)	444.1, <0.001	102.1, <0.001	56.2, <0.001	23.7, <0.001
Cohabiting	189 (24.5)	69 (6.7)	20 (2.8)		350 (49.9)	223 (17.0)	87 (8.6)				
Women											
Married	773 (78.3)	1,065 (94.5)	664 (97.5)	208.0, <0.001	492 (52.4)	1,277 (84.5)	1,066 (92.9)	558.0, <0.001	143.4, <0.001	65.2, <0.001	17.5, <0.001
Cohabiting	214 (21.7)	62 (5.5)	17 (2.5)		447 (47.6)	235 (15.5)	81 (7.1)				
Gender difference (*χ* ^2^, sig)	1.9, 0.166	1.3, 0.249	0.2, 0.688		0.8, 0.366	1.1, 0.300	1.8, 0.175				
**Gender balance of chores scale** ^b^											
Men											
Man does (almost) all chores	13 (1.7)	26 (2.5)	11 (1.6)		18 (2.6)	39 (3.0)	26 (2.6)				
Man does more chores	43 (5.6)	42 (4.1)	31 (4.4)		64 (9.1)	88 (6.7)	69 (6.8)				
Equally shared	75 (9.7)	65 (6.3)	34 (4.8)		125 (17.8)	159 (12.1)	99 (9.8)				
Woman does more chores	277 (35.9)	292 (28.3)	208 (29.6)	41.1, <0.001	248 (35.3)	419 (31.9)	288 (28.6)	58.3 <0.001	38.2, <0.001	48.0, <0.001	23.7, <0.001
Woman does (almost) all chores	364 (47.2)	607 (58.8)	419 (59.6)		247 (35.2)	608 (46.3)	526 (52.2)				
Women											
Man does (almost) all chores	7 (0.7)	8 (0.7)	9 (1.3)		14 (1.5)	22 (1.5)	23 (2.0)				
Man does more chores	28 (2.8)	24 (2.1)	17 (2.5)		58 (6.2)	72 (4.8)	60 (5.2)				
Equally shared	81 (8.2)	54 (4.8)	27 (4.0)		128 (13.6)	155 (10.3)	95 (8.3)				
Woman does more chores	308 (31.2)	279 (24.8)	188 (27.6)	36.6, <0.001	300 (31.9)	428 (28.3)	315 (27.5)	33.3, <0.001	37.6, <0.001	59.2, <0.001	24.8, <0.001
Woman does (almost) all chores	563 (57.0)	762 (67.6)	440 (64.6)		439 (46.8)	835 (55.2)	654 (57.0)				
Gender difference (*χ* ^2^, sig)	23.6, <0.001	29.2, <0.001	6.3, 0.180		25.8, <0.001	28.3, <0.001	7.1, 0.133				

	1991				2007				Date difference		
	Age 20–34 (born 1957–1971), *N* (%)	Age 35–49 (born 1942–1956), *N* (%)	Age 50–64 (born 1927–1941), *N* (%)	Age difference (*χ* ^2^, sig)	Age 20–34 (born 1973–1987), *N* (%)	Age 35–49 (born 1958–1972), *N* (%)	Age 50–64 (born 1943–57), *N* (%)	Age difference (*χ* ^2^, sig)	Age 20–34 (*χ* ^2^, sig)	Age 35–49 (*χ* ^2^, sig)	Age 50–64 (*χ* ^2^, sig)
**Couple employment**											
Men											
Both work	494 (64.0)	730 (70.7)	329 (46.8)		525 (74.8)	999 (76.1)	572 (56.7)				
Only the man works	170 (22.0)	206 (20.0)	158 (22.5)		119 (17.0)	229 (17.4)	198 (19.6)				
Only the woman works	37 (4.8)	51 (4.9)	82 (11.7)	166.6, <0.001	23 (3.3)	27 (2.1)	82 (8.1)	184.0, <0.001	22.2, <0.001	18.7, <0.001	17.9, <0.001
Neither work	71 (9.2)	45 (4.4)	134 (19.1)		35 (5.0)	58 (4.4)	156 (15.5)				
Women											
Both work	616 (62.4)	810 (71.9)	252 (37.0)		660 (70.3)	1,118 (73.9)	558 (48.6)				
Only the man works	249 (25.2)	209 (18.5)	163 (23.9)		217 (23.1)	267 (17.7)	218 (19.0)				
Only the woman works	41 (4.2)	56 (5.0)	88 (12.9)	342.2, <0.001	17 (1.8)	55 (3.6)	116 (10.1)	390.0, <0.001	22.7, <0.001	3.4, 0.333	23.8, <0.001
Neither work	81 (8.2)	52 (4.6)	178 (26.1)		45 (4.8)	72 (4.8)	255 (22.2)				
Gender difference (*χ* ^2^, sig)	2.9, 0.402	0.7, 0.864	16.4, 0.001		12.1, 0.007	6.7, 0.083	21.9, <0.001				
**Highest qualification**											
Men											
None	149 (19.3)	350 (33.9)	415 (59.0)		60 (8.5)	154 (11.7)	315 (31.3)				
Basic secondary school	309 (40.0)	271 (26.3)	136 (19.3)		218 (31.1)	447 (34.0)	229 (22.7)				
University entry level	162 (21.0)	216 (20.9)	72 (10.2)	271.6, <0.001	210 (29.9)	308 (23.5)	227 (22.7)	214.2, <0.001	67.1, <0.001	177.3, <0.001	145.8, <0.001
University/College	152 (19.7)	195 (18.9)	80 (11.4)		214 (30.5)	404 (30.8)	237 (23.5)				
Women											
None	152 (15.4)	514 (45.6)	464 (68.1)		65 (6.9)	166 (11.0)	447 (39.0)				
Basic secondary school	526 (53.3)	364 (32.3)	122 (17.9)		295 (31.4)	621 (41.1)	322 (28.1)				
University entry level	177 (17.9)	93 (8.3)	39 (5.7)	517.0, <0.001	260 (27.7)	301 (19.9)	149 (13.0)	497.5, <0.001	192.1, <0.001	431.8, <0.001	150.8, <0.001
University/College	132 (13.4)	156 (13.8)	56 (8.2)		319 (34.0)	424 (28.0)	229 (20.0)				
Gender difference (*χ* ^2^, sig)	32.7, <0.001	94.0, <0.001	17.2, 0.001		3.6, 0.306	15.4, 0.001	46.1, <0.001				
**Dependent children**											
Men											
No dependent children	303 (39.2)	306 (29.7)	619 (88.1)	613.0, <0.001	295 (42.0)	367 (28.0)	814 (80.8)	653.2, <0.001	1.2, 0.279	0.8, 0.366	16.2, <0.001
Any dependent children	469 (60.8)	726 (70.3)	84 (11.9)		407 (58.0)	946 (72.0)	194 (19.2)				
Women											
No dependent children	354 (35.9)	400 (35.5)	649 (95.3)	732.7, <0.001	368 (39.2)	435 (28.8)	1,013 (88.3)	990.0, <0.001	2.3, 0.132	13.5, <0.001	25.2, <0.001
Any dependent children	633 (64.1)	727 (64.5)	32 (4.7)		571 (60.8)	1,077 (71.2)	134 (11.7)				
Gender difference (*χ* ^2^, sig)	2.1, 0.146	8.3, 0.004	23.7, <0.001		1.3, 0.247	0.2, 0.630	23.8, <0.001				

^a^Basic distributions and analyses of group differences based on unweighted data

^b^Based on responses to four items asking who did particular household chores, each scored −1 if mostly done by the man, +1 if mostly the woman and 0 if shared or done by someone else. In this collapsed scale, ‘man does (almost) all’ = scores −4 or −3, ‘man does more’ = scores −1 or −2, equally shared = score 0, ‘woman does more’ = scores 1 or 2, ‘woman does (almost) all’ = scores 3 or 4

### Measures

Psychological distress was measured via the 12-item General Health Questionnaire (GHQ-12) [44] which has been extensively used as a screening instrument in large population surveys of psychological morbidity [45, 46]. The GHQ is a brief self-report instrument for the detection of mental disorders in the community and among primary care patients. It was designed as a measure of state; thus respondents are asked to consider ‘the past few weeks’. The 12 items focus on both inability to carry out normal functions (e.g. ‘been able to enjoy your normal day-to-day activities’; ‘been able to concentrate on whatever you’re doing’) and the emergence of distressing symptoms (e.g. ‘felt constantly under strain’; ‘been losing confidence in yourself’). Each item includes four answer options ranging from ‘more than usual’ to ‘much less than usual’ (normal functions) or from ‘not at all’ to ‘much more than usual’ (distressing symptoms). Although the measure can be used categorically (those in the population scoring above specified cut-offs), it can also be scored as a Likert scale (0–1–2–3, resulting range 0–36), as we have done here, since we are interested in associations along the full spectrum of psychological distress. The GHQ is one of the most thoroughly tested of all health measures, and validation studies have been undertaken in many different countries [47–49]. Its psychometric properties are well established, with previous studies of the GHQ-12 reporting split-half reliability of 0.83 and alpha coefficients ranging from 0.82 to 0.90 [46].

To measure traditional GRAs, BHPS respondents were asked to indicate their level of agreement (five-point scale, strongly agree—strongly disagree) with six statements. Three represented more traditional opinions (‘a pre-school child is likely to suffer if his or her mother works’; ‘all in all, family life suffers when the woman has a full-time job’; ‘a husband’s job is to earn money; a wife’s job is to look after the home and family’) and three more egalitarian opinions (‘a woman and her family would all be happier if she goes out to work’; ‘both the husband and wife should contribute to the household income’; ‘having a full-time job is the best way for a woman to be an independent person’). The egalitarian statements were reverse coded so that a higher score indicated more traditional values, and a ‘traditionalism’ scale (possible range 1–5) was constructed using the mean of the scores for the six statements. This method is identical [27] or very similar [13] to that used in previous studies of GRAs within the BHPS and also to other studies of GRAs conducted in the US, UK and elsewhere in Europe [7, 8, 40, 50–52]. The internal consistency of the traditionalism scale (alpha coefficientss), calculated for males and females in each of the three age groups at each of the two dates, ranged from alpha = 0.68 (20- to 34-year-old males in 2007) to alpha = 0.75 (20- to 34-year-old males in 1991). In other studies which provide these details, the alpha values for traditionalism scales also fall around 0.70 [7, 8, 13, 27].

Three ‘couple role’ variables were included. Marital status was categorised as married vs. cohabiting. To investigate the gender-division of household chores, we created a gender-balance of daily chores scale based on responses to four items asking who did the grocery shopping, cooking, washing/ironing and cleaning. Studies have found these chores to be some of the most time-consuming [53] and ‘low-control’ in the sense of being routine and unavoidable [5]. (Note that we did not include items relating to childcare because they were not applicable to all respondents.) Each item was scored -1 if the man mostly did that chore, +1 if the woman did it and 0 if the chore was shared or done by someone else. Positive values on the resulting scale (range −4 to +4), therefore, represent more chores being performed by the woman. (Previous studies suggest that men tend to over-report their involvement in chores [1, 8]. Where both couple members had responded, it was possible to determine their agreement in respect of who did each chore: if the woman responded ‘mostly self’ and the man ‘mostly spouse/partner’ this was agreement; however, if the man also responded ‘mostly self’, this was disagreement. Analyses [not shown] of levels of agreement found these were 80 % for ‘who does the cleaning’, 84 % for cooking and 85 % for grocery shopping and washing/ironing.) Finally, information on economic activity allowed couple employment to be categorised as both couple members employed (both full- and part-time paid employment or self-employed); only the man; only the woman; or neither (both couple members unemployed, retired, family care, full time student, long-term sick/disabled, maternity leave, government training scheme, other).

To account for educational level we used highest academic qualification, categorised as none; ‘O’ level/CSE or equivalent (basic secondary school qualifications); ‘A’ level or equivalent (secondary school qualifications required for university entrance); or university/college. Our analyses also included the BHPS-derived variable dependent children in the household, defined as those aged under 16, or aged 16–18 and in school or non-advanced further education, not married and living with a parent.

### Analysis

All analyses were carried out in Stata 11.1. Frequencies and descriptive statistics were obtained for the measures by gender, age group and date, with differences (gender differences for each age group at each date; differences between the three age groups for men and women at each date; and differences between the two dates for men and women in each age group) in proportions via Chi square and in means via bivariable linear regression (*t*-statistic).

Although a small proportion of respondents (17 %) participated in both 1991 and 2007, the fact that these dates were 16 years apart meant that no respondent was in the same age-group at the two dates. Since analyses (described below) suggested very few differences between 1991 and 2007 in the associations which either traditionalism or GHQ had with ‘couple roles’, the decision was made to combine data from the two dates separately for each age-group and to focus on differences between the three age groups, for which there was more evidence.

To explore the relationship between traditionalism and marital status, the gender-balance of chores, couple employment, highest qualifications and dependent children in the household, a series of bivariable linear regression models were run separately for each of six gender and age sub-groups (i.e. men and women aged 20–34, 35–49 and 50–64), having combined the data from 1991 to 2007. Within each age group, regression models also examined whether associations differed for men and women (interactions with gender) and between 1991 and 2007 (interactions with date). In order to determine whether the separate regression coefficients obtained for the three age-groups differed from each other, the Stata ‘seemingly unrelated estimation’ (suest) procedure was used. This procedure is able to account for the fact that the separate regressions may feature (some of) the same respondents. Thus, to obtain the row of figures showing associations between marital status and traditionalism in Table 3, we ran the following: bivariable regressions of marital status on the traditionalism score for both men and women in each of the three age-groups; regressions including marital status, gender and the marital status by gender interaction on the traditionalism score for each of the three age-groups; regressions including marital status, date and the marital status by date interaction on the traditionalism score for both men and women in each of the three age-groups; and ‘seemingly unrelated estimation’ to compare the regression coefficients in 20- to 34-year-olds vs. 35- to 49-year-olds, 20- to 34-year-olds vs. 50- to 64-year-olds, and 35- to 49-year-olds vs. 50- to 64-year-olds, for both men and women (detailed in Supplementary Table 2). Similar bivariable linear regression models then examined associations between GHQ and date, traditionalism, the three ‘couple role’ variables, qualifications and dependent children in each of the six gender and age sub-groups, also identifying differences according to gender, date and age-group (Table 4 and Supplementary Table 3). These were followed by multivariable linear regression models to examine the mutually adjusted associations between GHQ and date, traditionalism, the ‘couple role’ variables, qualifications and dependent children in each of the six sub-groups (Table 5).

Finally, in order to examine whether levels of psychological distress were higher when attitudes conflicted with actual roles, additional multivariable models also included interactions between the traditionalism score and each of the three ‘couple role’ variables (Supplementary Table 4). To further investigate any significant interactions, separate analyses were conducted for those in the lowest and highest tertiles of traditionalism (representing the least and most traditional individuals) in each sub-group (Table 6).

Given the inclusion of the booster samples in 2007 and the differential response to each survey wave, cross-sectional inverse probability weights [43] have been applied to all analyses (unless indicated) of the two separate waves employed here. These weights ensure each wave is representative of the general population in those years, but very slightly reduce the size of the 2007 sample (which includes booster samples that were proportionately oversampled originally, and so are down-weighted).

## Results

### Descriptive results

In Table 1, which describes the samples and shows the significance of differences according to gender, age-group and date, perhaps the most striking finding is differences in levels of cohabitation according to both date and age-group: in our sample around 3 % of 50- to 64-year-olds in 1991 were cohabiting, compared with half of 20- to 34-year-olds in 2007. Differences according to age group were evident for all five variables (marital status, gender-balance of chores, couple employment, highest qualification and dependent children) among both men and women and at both dates (all significant *p* < 0.001). Thus, among the 50- to 64-year-olds at both dates, levels of cohabitation were lowest, the woman was more likely to do (almost) all the chores, the proportions reporting that only the woman was in paid employment or that neither couple member worked were highest, educational qualifications were lowest and the proportion reporting any dependent children in the household was much lower than in either of the other two age groups. Differences according to date were also evident for all five variables among both men and women in every age group (all significant *p* < 0.001) with just a few exceptions. The exceptions included couple employment (no differences between 1991 and 2007 among 35- to 49-year-old women, while in all other groups the proportions reporting both couple members worked were higher at the later date) and dependent children in the home (no differences between 1991 and 2007 for 20- to 34-year-old men and both 20- to 34 and 35- to 49-year-old women, while in older groups the proportion with dependent children was higher at the later date).

At neither date, and in none of the three age groups was there a gender difference in marital status; however, in both the 20- to 34-year-old and 35- to 49-year-old groups, reports that chores were shared or done by the man were significantly more likely to made by men than women (similar gender differences among the oldest age groups were not significant at either date). While levels of chore sharing were somewhat higher among the youngest age groups and at the later date, even among 20- to 34-year-old respondents in 2007, around 70 % of men and women reported that chores were done more by the woman, 18 % of men and 14 % women that they were equally shared, and only 12 % men and 8 % women that they were done more by the man. Among 50- to 64-year-olds at both dates, greater proportions of men reported that both couple members worked and greater proportions of women reported that neither did, while in 2007 the proportion reporting only the man worked was higher among women. There were significant gender differences in qualification levels in every age group in 1991 and the mid and oldest age groups in 2007 (all *p* ≤ 0.001, all higher qualifications among men). Finally, while there were no gender differences in reports of dependent children in the household among 20- to 34-year-old, 35- to 49-year-old men in 1991 and 50- to 64-year-old men at both dates were more likely than women to report living with dependent children.

### Group differences in traditionalism

The first set of analyses in relation to our hypotheses examined whether traditionalism was lower: among women; among younger people; at the later of the two dates; among those in ‘less traditional’ heterosexual couple relationships; and among those with higher qualifications.

Table 2 shows traditionalism and GHQ likert scores in 1991 and 2007, both overall and by gender- and age-band. Traditionalism scores were significantly lower among women than men in both 1991 (gender difference *t* = −6.7, *p* < 0.001) and 2007 (*t* = −5.4, *p* < 0.001), with somewhat greater gender differences among older age-groups. Among both men and women, traditionalism increased significantly with age at both dates and was higher at the earlier date in each age group.

**Table 2:** Mean (standard error) traditionalism and GHQ Likert scores for men and women in each age group at both dates with tests for significances of: gender differences for each age group at each date; differences between the three age groups for men and women at each date; and differences between the two dates for men and women in each age group

	1991			2007			Date difference	
	Men [mean (SE)]	Women [mean (SE)]	Gender diff, *t* (sig)	Men [mean (SE)]	Women [mean (SE)]	Gender diff, *t*, sig	Men (*t*, sig)	Women (*t*, sig)
Traditionalism								
Overall	2.97 (0.01)	2.85 (0.01)	−6.7, <0.001	2.89 (0.01)	2.79 (0.01)	−5.4, <0.001		
Aged 20–34	2.83 (0.03)	2.75 (0.02)	−2.3, 0.023	2.73 (0.03)	2.67 (0.03)	−1.7, 0.093	−2.6, 0.010	−2.3, 0.023
Aged 35–49	2.98 (0.02)	2.87 (0.02)	−4.0, <0.001	2.87 (0.02)	2.77 (0.02)	−3.5, <0.001	−3.9, <0.001	−3.6, <0.001
Aged 50–64	3.09 (0.02)	2.95 (0.02)	−4.4, <0.001	2.98 (0.02)	2.87 (0.02)	−3.6, <0.001	−3.2, 0.001	−2.4, 0.018
Age difference (*t*, sig)								
35–49 vs. 20–34	4.8, <0.001	4.0, <0.001		4.1, <0.001	2.9, 0.003			
50–64 vs. 20–34	7.7, <0.001	6.1, <0.001		7.3, <0.001	5.8, <0.001			
GHQ Likert								
Overall	10.20 (0.09)	11.06 (0.09)	6.7, <0.001	10.49 (0.12)	11.52 (0.12)	6.1, <0.001		
Aged 20–34	9.80 (0.16)	11.28 (0.15)	6.6, <0.001	9.88 (0.21)	10.95 (0.21)	3.6, <0.001	0.3, 0.784	−1.3, 0.200
Aged 35–49	10.58 (0.15)	11.22 (0.14)	3.2, 0.002	10.84 (0.19)	11.73 (0.19)	3.3, 0.001	1.1, 0.271	2.1, 0.034
Aged 50–64	10.06 (0.17)	10.53 (0.18)	1.9, 0.058	10.37 (0.19)	11.58 (0.20)	4.3, <0.001	1.2, 0.228	3.8, <0.001
Age difference (*t*, sig)								
35–49 vs. 20–34	3.5, <0.001	−0.3, 0.771		3.4, 0.001	2.7, 0.007			
50–64 vs. 20–34	1.1, 0.275	−3.2, 0.002		1.7, 0.082	2.1, 0.032			

Table 3 shows the unadjusted relationships that the three ‘couple role’ variables, qualifications and dependent children in the household had with traditionalism, among men and women in each age band. Data from the two dates were combined since, as the right-hand section of the table shows, additional analyses demonstrated very few interactions with date (further details available in Supplementary Table 2). In all gender and age sub-groups, traditionalism was significantly lower among cohabiting than married respondents and traditionalism was positively associated with the female doing more chores. However, as the far right-hand section of the table shows, the association between traditionalism and the gender-balance of chores was significantly lower among 50- to 64-year-old men and women than those in the younger two age-groups. When compared with couple members from households where both were employed, traditionalism was significantly higher when only the man was employed (particularly in the younger two age groups) and when neither couple member worked (this relationship was weaker, although still significant, among women in the oldest age-group). Among women in all three age-groups, traditionalism was significantly lower among those with university/college education compared with those who had no qualifications; similar trends for men were non-significant. Finally, among both 20- to 34-year-old men and, to a lesser extent, 35- to 49-year-old men and women, traditionalism was higher among those with dependent children. However, there were no significant associations between traditionalism and dependent children in the oldest age-group. The pattern of associations was very similar for men and women, with only three significant interactions with gender (among the 20- to 34-year-olds, the positive association between traditionalism and dependent children was stronger in women; among the 35- to 49-year-olds, lower levels of traditionalism for those with university/college qualifications compared with none, was only significant in women; and among 50- to 64-year-olds, levels of traditionalism were increased to a greater extent among men than women when neither couple member worked compared with when both worked).

**Table 3:** Unadjusted relationships with Traditionalism score: coefficients and significance for men and women, and significance of gender difference in each age group; indication of any significant differences between the two dates; and indication of any significant differences between the three age groups (further details in Supplementary Table 2)

	Age 20–34					Age 35–49					Age 50–64					Sig (*p* < 0.05) date difference		Sig (*p* < 0.05) age-group difference	
	Men		Women		Gender diff (sig)	Men		Women		Gender diff (sig)	Men		Women		Gender diff (sig)	Men	Women	Men	Women
	Coef	Sig	Coef	Sig		Coef	Sig	Coef	Sig		Coef	Sig	Coef	Sig					
Marital status (married)																			
Cohabiting	−0.22	<0.001	−0.25	<0.001	0.590	−0.16	<0.001	−0.17	<0.001	0.795	−0.17	0.005	−0.18	0.014	0.892				
Gender balance of chores scale (higher = woman does more)	0.08	<0.001	0.09	<0.001	0.562	0.06	<0.001	0.07	<0.001	0.452	0.04	<0.001	0.03	0.002	0.640		^a^	^e, f^	^e, f^
Couple employment (both work)																			
Only the man works	0.40	<0.001	0.45	<0.001	0.325	0.42	<0.001	0.42	<0.001	0.987	0.27	<0.001	0.20	<0.001	0.266	^a^		^e, f^	^e, f^
Only the woman works	−0.10	0.264	0.01	0.906	0.368	0.10	0.171	−0.08	0.192	0.059	−0.05	0.327	−0.06	0.288	0.958				
Neither work	0.23	0.001	0.42	<0.001	0.062	0.27	<0.001	0.31	<0.001	0.634	0.28	<0.001	0.13	0.002	0.012				^e, f^
Highest qualification (none)																			
Basic secondary school	0.00	0.937	−0.05	0.351	0.492	0.02	0.695	−0.02	0.601	0.521	−0.03	0.434	0.01	0.885	0.499				
University entry level	−0.04	0.508	−0.14	0.028	0.274	0.02	0.710	0.01	0.814	0.937	0.03	0.608	0.08	0.095	0.408				^e^
University/College	−0.07	0.259	−0.20	0.001	0.132	−0.07	0.104	−0.20	<0.001	0.028	−0.07	0.151	−0.17	<0.001	0.140	^a^	^a^		
Children in household (none)																			
Any dependent children	0.21	<0.001	0.33	<0.001	0.009	0.11	<0.001	0.08	0.006	0.556	−0.07	0.192	−0.02	0.768	0.494	^a, c^		^d, e, f^	^d, e, f^
Weighted *N*	1445		1,884		3,329	2,316		2,611		4,927	1,706		1,822		3,528				

^a^Significant interaction with date in youngest cohorts

^b^Significant interaction with date in middle cohorts

^c^Significant interaction with date in oldest cohorts

^d^Significant difference between youngest and middle cohorts

^e^Significant difference between youngest and oldest cohorts

^f^Significant difference between middle and oldest cohorts

Overall, this first set of analyses shows lower traditionalism among women, younger people, those taking part in the survey at the more recent date and both men and women in ‘less traditional’ relationships and households. Although some associations with traditionalism differed between age groups, there was very little evidence of different associations in either men compared with and women or in 1991 compared with 2007.

### Associations with psychological distress

The next set of analyses examined the associations which traditionalism and the three ‘couple role’ variables had with psychological distress. Education and the presence of dependent children in the household were also included in the models; education because of its associations with GRAs, roles [8] and psychological distress [42], and dependent children because of their assumed effect on household chores. Table 4, therefore, shows the unadjusted relationships which traditionalism, each of the ‘couple role’ variables, highest qualification and dependent children had with GHQ Likert score. Again, results are shown for men and women in each age band and data from the two dates were combined since additional analyses showed almost no differences according to date (see right-hand section of Table 4 and further details available in Supplementary Table 3). Unadjusted associations between GHQ score and date are shown: among 35- to 49-year-old and, even more so, 50- to 64-year-old women, psychological distress was significantly higher in 2007 than in 1991.

**Table 4:** Unadjusted relationships with GHQ Likert score: coefficients and significance for men and women, and significance of gender difference in each age group; indication of any significant differences between the two dates; and indication of any significant differences between the three age groups (further details in Supplementary Table 3)

	Age 20–34					Age 35–49					Age 50–64					Sig (*p* < 0.05) date difference		Sig (*p* < 0.05) age-group difference	
	Men		Women		(Gender diff, sig)	Men		Women		(Gender diff, sig)	Men		Women		(Gender diff, sig)	Men	Women	Men	Women
	Coef	Sig	Coef	Sig		Coef	Sig	Coef	Sig		Coef	Sig	Coef	Sig					
Date (1991)																			
2007	0.07	0.784	−0.34	0.200	0.274	0.27	0.271	0.50	0.034	0.486	0.31	0.228	1.05	<0.001	0.051	N/A	N/A		^d, e^
Traditionalism score	0.44	0.033	0.90	<0.001	0.099	0.67	0.001	0.55	0.007	0.669	0.56	0.014	0.79	0.003	0.521				
Marital status (married)																			
Cohabiting	0.14	0.610	−0.09	0.750	0.559	0.18	0.668	0.94	0.070	0.257	0.12	0.830	0.07	0.901	0.955				
Gender balance of chores scale (higher = woman does more)	−0.05	0.509	0.13	0.121	0.116	−0.00	1.000	−0.18	0.030	0.085	0.05	0.468	−0.22	0.025	0.026	^c^			^d, e^
Couple employment (both work)																			
Only the man works	0.68	0.022	0.88	0.004	0.635	0.07	0.830	0.57	0.122	0.303	−0.23	0.436	0.38	0.352	0.226				
Only the woman works	3.16	<0.001	2.65	0.001	0.653	4.66	<0.001	0.87	0.220	0.001	1.36	0.008	−0.29	0.504	0.014	^a^		^d, f^	^e^
Neither work	2.91	<0.001	2.76	<0.001	0.849	4.99	<0.001	3.61	<0.001	0.337	1.95	<0.001	0.53	0.168	0.018			^f^	^e, f^
Highest qualification (none)																			
Basic secondary school	−0.75	0.079	−1.19	0.003	0.444	−0.16	0.645	−0.03	0.930	0.776	−0.56	0.125	−0.26	0.466	0.552				
University entry level	−0.77	0.073	−1.14	0.013	0.554	−0.19	0.595	−0.15	0.695	0.949	−0.97	0.007	0.63	0.259	0.016				^e, f^
University/College	−0.68	0.132	−1.42	0.002	0.262	0.17	0.637	−0.46	0.188	0.208	−0.98	0.011	−0.91	0.019	0.907			^f^	^d^
Children in household (none)																			
Any dependent children	0.57	0.032	0.72	0.006	0.671	−0.22	0.452	0.05	0.851	0.503	0.95	0.017	0.42	0.473	0.457			^d, f^	
Weighted *N*	1,445		1,884		3,329	2,316		2,611		4,927	1,706		1,822		3,528				

^a^Significant interaction with date in youngest cohorts

^b^Significant interaction with date in middle cohorts

^c^Significant interaction with date in oldest cohorts

^d^Significant difference between youngest and middle cohorts

^e^Significant difference between youngest and oldest cohorts

^f^Significant difference between middle and oldest cohorts

In all gender and age sub-groups, higher traditionalism was associated with poorer mental health; all associations between traditionalism and GHQ score were significant and positive. Marital status was not related to GHQ score. However, in both 35- to 49-year-old and 50- to 64-year-old women there was an association between the gender-balance of chores and GHQ, with lower GHQ scores among those who reported doing more chores themselves. The far right-hand section of Table 4 shows that this contrasts with a non-significant association in the opposite direction among 20- to 34-year-old women. Couple employment showed by far the most marked associations with GHQ. When only the woman worked (compared with when both couple members did), GHQ scores were significantly higher among men of all ages, but particularly 35- to 49-year-olds; they were also significantly higher among 20- to 34-year-old women although not women in either of the other two age groups. When neither couple member worked, GHQ scores were significantly higher among men of all ages (although the association was strongest among 35- to 49-year-olds and weakest among 50- to 64-year-olds) and among women in the two younger age groups. GHQ scores reduced with increasing qualifications in most sub-groups, although the difference between those with none compared with university/college level qualifications was only significant among 20- to 34-year-old women and 50- to 64-year-old men and women. Finally, GHQ scores were higher for those with dependent children compared with none in both younger and older age groups (significant among all except older women), but there were no associations between dependent children and GHQ score among 35- to 49-year-olds. As the table shows, there were a number of significant interactions with gender, particularly in the oldest age group (GHQ score negatively associated with the woman doing more chores in women only and with university entry level qualifications in men only, and positively associated with only the woman working and with neither couple member working, in men only).

The mutually adjusted relationships which date, traditionalism, each of the ‘couple role’ variables, highest qualification and dependent children had with GHQ Likert score among men and women in each age band are shown in Table 5. Mutual adjustment increased the strength of the relationship with date, resulting in significantly higher scores in 2007 compared with 1991 for both men and women in the 35- to 49-year-old and 50- to 64-year-old age groups. Adjustment weakened associations between traditionalism and GHQ score in the youngest age group, but had no impact in the two older age groups. Adjustment also had very little impact on associations between GHQ score and both the gender-balance of chores and couple employment. However, it reduced relationships between GHQ score and education in the 20- to 34-year-old women and between GHQ score and dependent children in 20- to 34-year-old men and women, to non-significance.

**Table 5:** Mutually adjusted relationships with GHQ likert score: coefficients and significance for men and women in each age group

	Age 20–34				Age 35–49				Age 50–64			
	Men		Women		Men		Women		Men		Women	
	Coeff	Sig	Coeff	Sig	Coeff	Sig	Coeff	Sig	Coeff	Sig	Coeff	Sig
Date (1991)												
2007	0.26	0.358	−0.13	0.664	0.49	0.046	0.59	0.021	0.75	0.007	1.21	<0.001
Traditionalism score	0.37	0.087	0.69	<0.001	0.67	0.002	0.54	0.010	0.53	0.023	0.77	0.004
Marital status (married)												
Cohabiting	0.06	0.846	0.16	0.598	−0.04	0.918	0.86	0.101	0.08	0.886	0.10	0.861
Gender balance of chores scale (higher = woman does more)	−0.04	0.618	0.07	0.454	0.05	0.496	−0.16	0.051	0.14	0.075	−0.25	0.013
Couple employment (both work)												
Only the man works	0.52	0.112	0.44	0.187	−0.18	0.569	0.40	0.298	−0.44	0.150	0.42	0.318
Only the woman works	3.17	<0.001	2.64	0.001	4.91	<0.001	0.83	0.243	1.55	0.003	−0.37	0.409
Neither work	2.75	<0.001	2.18	<0.001	5.10	<0.001	3.24	<0.001	1.83	<0.001	0.37	0.334
Highest qualification (none)												
Basic secondary school	−0.24	0.573	−0.74	0.067	0.08	0.819	−0.14	0.651	−0.56	0.121	−0.55	0.133
University entry level	−0.15	0.730	−0.47	0.323	0.20	0.568	−0.32	0.432	−1.07	0.003	0.31	0.579
University/College	0.07	0.876	−0.58	0.249	0.72	0.042	−0.51	0.168	−1.14	0.003	−1.21	0.003
Children in household (none)												
Any dependent children	0.27	0.389	0.16	0.620	−0.36	0.203	0.19	0.525	1.13	0.004	0.40	0.498
Weighted *N*	1,445		1,884		2,316		2,611		1,706		1,822	

Overall, these analyses suggest that psychological distress was higher among those with more traditional GRAs. There was no evidence of lower psychological distress in households where men took on some of the chores; indeed, the opposite was the case among mid and older age women. In all gender and age sub-groups apart from the oldest women, psychological distress was most clearly associated with the man not working (i.e. only the woman worked or neither couple member worked).

### Is psychological distress higher when attitudes and roles conflict?

Our third set of analyses examined whether levels of psychological distress were higher when attitudes conflicted with actual roles. In order to do this, additional multivariable analyses were conducted for each of the six gender and age sub-groups, entering all variables (as Table 5) together with the interactions between traditionalism and each of the three ‘couple role’ variables (marital status, gender-balance of chores and household work). The results of the interaction analyses are shown in Supplementary Table 4. Among men, none of the possible 15 interactions were significant at *p* < 0.10; however, among women five were significant at this level.

In order to further investigate the interactions found for women, separate analyses were conducted for those in the lowest and highest tertiles of traditionalism (representing women who we describe as ‘egalitarian’ and ‘traditional’) in each age sub-group. These analyses examined the mutually adjusted associations which each of the three couple role variables, date, qualifications and dependent children had with GHQ score. Table 6 shows the results; the five boxes indicate significant (*p* < 0.10) interactions with traditionalism.

**Table 6:** Mutually adjusted relationships with GHQ likert score—‘egalitarian’ and ‘traditional’ women in each age group

	Age 20–34				Age 35–49				Age 50–64			
	Least traditional tertile (‘egalitarian’ women)		Most traditional tertile (‘traditional’ women)		Least traditional tertile (egalitarian’ women)		Most traditional tertile (‘traditional’ women)		Least traditional tertile (egalitarian’ women)		Most traditional tertile (‘traditional’ women)	
	Coeff	Sig	Coeff	Sig	Coeff	Sig	Coeff	Sig	Coeff	Sig	Coeff	Sig
Date (1991)												
2007	0.03	0.946	−0.40	0.387	0.19	0.651	0.02	0.968	1.38	0.014	1.33	0.009
Marital status (married)												
Cohabiting	**−0.76**	**0.089**	**1.14**	**0.061**	1.03	0.166	1.68	0.141	0.05	0.956	−0.63	0.601
Gender balance of chores scale (higher = woman does more)	−0.04	0.776	0.09	0.514	**0.09**	**0.488**	**−0.34**	**0.028**	−0.05	0.806	−0.28	0.072
Couple employment (both work)												
Only the man works	**1.72**	**0.028**	**−0.11**	**0.810**	1.76	0.050	0.36	0.493	**2.37**	**0.027**	**−0.79**	**0.186**
Only the woman works	**1.70**	**0.216**	**3.97**	**0.003**	1.03	0.368	0.59	0.588	−0.82	0.204	−0.40	0.700
Neither work	3.56	<0.001	1.84	0.023	0.94	0.356	2.67	0.040	−0.12	0.867	0.19	0.772
Highest qualification (none)												
Basic secondary school	−1.01	0.114	−0.31	0.650	−0.21	0.643	−0.79	0.105	−0.41	0.578	−0.59	0.320
University entry level	−0.42	0.560	−0.15	0.860	−0.38	0.575	0.10	0.890	−1.48	0.088	1.05	0.306
University/college	−0.47	0.538	−0.76	0.367	−0.98	0.079	0.04	0.958	−1.32	0.070	−0.80	0.289
Children in household (none)												
Any dependent children	−0.23	0.625	1.03	0.073	0.90	0.050	−0.16	0.761	−0.11	0.911	0.57	0.536

Bold = significant interactions with (continuous) traditionalism (i.e. those shown as *p* < 0.100 on Supplementary Table 4)

As Table 5 shows, each of these results ‘fits’ the hypothesis of greater psychological distress when attitudes and roles conflict. Thus, among 20- to 34-year-olds, ‘egalitarian’ cohabiters had lower, while ‘traditional’ cohabiters had higher GHQ scores than married women. In other words, among these young women in less traditional (cohabiting) households, psychological distress was somewhat lower among those with ‘egalitarian’ GRAs (the group for whom attitudes and role were consistent) and higher among those with ‘traditional’ GRAs (conflicting attitudes and role). Among the 35- to 49-year-olds, doing more chores oneself (rather than sharing them, or the man doing more) was associated with a significantly lower GHQ score among ‘traditional’, but not ‘egalitarian’ women. (Note also a similar pattern among the 50- to 64-year-old women, although this interaction was non-significant.) The remaining three of these interaction results related to couple employment. Thus, among both 20- to 34 and 50- to 64-year-olds, GHQ scores were significantly higher among ‘egalitarian’, but not ‘traditional’ women in households where the man was the sole breadwinner, compared with women in households where both couple members worked. Further, among the 20- to 34-year-olds, GHQ scores were significantly higher among ‘traditional’, but not egalitarian women in households where they themselves were the sole breadwinner, compared with women in households where both couple members worked.

## Discussion

Our analyses, based on UK samples of younger, middle and older working-age men and women in 1991 and 2007, aimed to examine levels of traditionalism and associations between GRAs, ‘couple roles’ and psychological distress. Expectations of lower traditionalism among women, younger people, at the later of the two dates, those in ‘less traditional’ heterosexual couple relationships (cohabiting, the man doing/sharing chores, the woman employed and/or the man not employed, no children) and those with more qualifications were, by and large, upheld. Previous studies suggest much of the generational difference is explained by educational level and, for females, labour market experience and marital status [8, 40]. Other authors suggest relationships between female GRAs and their labour market participation [27], family formation [54] and division of household responsibilities [35] are reciprocal, but that this is less the case for men, for whom there are more pressures to remain in full-time employment [55]. Given this, we might have expected to find stronger associations between GRAs and our ‘couple employment’ measure for women. However, this was not the case, and it should also be recognised that for many women, as well as men, labour market and lifestyle choices are subject to structural and normative constraints [15, 52, 56].

Gender traditionalism was positively associated with psychological distress in both men and women; analysis of a BHPS sample of older married couples found similar associations, but in women only [13]. Previous authors have suggested this relationship is the result of the benefits felt by all from there being more equal sharing of power and status [8] or the possibility that those with traditional views feel at odds with contemporary society [14]. The results of a cross-cohort, cross-national analysis of changes in the traditionality of actual female roles are at variance with these ideas. This study found that despite lower traditionality in younger cohorts across both developed and developing countries, gender differences in anxiety disorders and almost all mood disorders remained stable. However, there was one exception: excess prevalence of major depressive disorder in women decreased as female gender roles became more egalitarian, which the study’s authors interpret as meaning that increasing female opportunities lead to improved female mental health [6].

There is evidence that, on average, mental health is better among married than unmarried people, particularly for men [57]. However, studies do not generally find the effects extend to those who cohabit [58, 59], a result which has been attributed to the poorer quality of their relationships [60, 61]. It is, therefore, somewhat puzzling that we did not find significant differences in GHQ scores between respondents who were cohabiting compared with married. This might reflect the continuing erosion of a distinction which held deep social significance until the mid twentieth century at least, particularly when a couple had children. If so, we might have expected a different pattern of associations at the two dates, or when the youngest and oldest age-groups were compared, given increasing rates (and normalisation) of cohabitation. However, there was no evidence of this either. Presumably if we had included a measure of relationship quality, we would have found this to be associated with psychological distress [62].

Several, but by no means all, previous studies have found lower well-being among both men and women who spend more time on housework, who share household responsibilities less equally and/or perceive them to be shared less equally [8, 19, 20]. A number of hypotheses have been proposed to explain why shared tasks might benefit both men and women, including the ideas that equitable relationships promote well-being and that the symbolic meaning of men’s contribution to the household is important [34]. We found no association between the gender-balance of household chores and psychological distress in men. In addition, and contrasting with a trend towards increasing distress among women in the youngest age-group who did more chores themselves, women in the mid and oldest age-groups who reported doing more chores had lower levels of distress. Importantly, however, our subsequent analyses, discussed later, suggested these results were driven by associations among women with more traditional GRAs.

Among men, particularly those of mid working age, not being in paid employment was associated with psychological distress, consistent with previous BHPS analyses [41, 63] and a substantial amount of other evidence [29, 30]. However, although women in the two younger age groups living in households where neither they nor their partner worked had higher levels of psychological distress (perhaps as a result of the associated poverty [28]), there was less evidence that women’s psychological distress was higher when they themselves were not in paid employment, but their partner was. Further, in the oldest age-group, psychological distress was raised among men who were not in paid employment, but not among women whose partners did not work. This might have been because the partners of these women were slightly older than themselves and thus defined as ‘retired’ rather than ‘unemployed’. More generally, stronger effects of unemployment on the mental health of men than women have been attributed to links between paid employment and masculine identity and the associated greater stigmatisation of male unemployment, together with the fact that because men generally earn more money than women, unemployed men tend to receive less financial support from working wives or partners than unemployed women receive from working husbands [30]. Indeed, for some women, their household’s economic circumstances will have allowed them to choose not to enter the labour market. In relation to this, it is interesting that one study found unhappily married wives were more likely to move into full-time employment than happily married ones [64].

This notion of choice leads to our final set of analyses, interactions conducted to see whether psychological distress might be greater when GRAs (as more traditional or egalitarian) conflicted with actual household and paid work roles. This was not the case for men. However, for women, there was some rather weak evidence that GRAs-role consistency might matter, particularly in respect of couple employment. It has been argued that GRAs act as ‘a kind of lens’ through which women view the division of household labour (p. 1031) [35] and, it might be added, other aspects of their lives as well. In line with this, we found evidence that a ‘traditional’ gender-balance of household chores was related to lower levels of psychological distress in mid and older working age women with ‘traditional’ GRAs. We might ask why ‘egalitarian’ women did not show increased psychological distress when faced with a ‘traditional’ household chores balance. The reason might be that such a situation is simply accepted. There is evidence that even among young, unmarried, undergraduates, females continue to expect inequity in the division of household labour and child-care [65] and that although women generally do more household chores they tend not to perceive this as unfair [53], perhaps because they compare themselves with other households with a similar or less equal gender-division, feel they are more competent to do the work, or more valued by it [66, 67].

In respect of couple employment, there was evidence in two age-groups that psychological distress was higher among those women who had more egalitarian attitudes but were in a household where the man was the sole breadwinner, and/or among those with more traditional attitudes who were themselves the sole breadwinner. These results are each in line with the notion that while women with more egalitarian attitudes might feel confined by the traditional ‘housewife’ role, women with more traditional attitudes are not, but are instead more psychologically distressed by the ‘breadwinner’ role. However, we would have more confidence in this conclusion had we seen consistent interactions reflecting greater psychological distress among both women with more egalitarian attitudes in male breadwinner households and women with more traditional attitudes in female breadwinner households, which we did not. We would also have been more confident had we seen similar interactions among all age groups, which we did not. It is likely that a number of other factors will have affected these relationships, including whether the woman’s husband or partner held traditional or egalitarian GRAs (although previous studies, including analyses of the BHPS find moderate correlations between the GRAs of men and their wives or partners [10, 68]), the nature of the woman’s employment (in particular whether full- or part-time) and her other roles. The authors of one paper which found no evidence that lack of fit between attitudes and behaviour impacted on marital dissatisfaction suggested that such inconsistencies may be tolerated as ‘unavoidable consequences of individual circumstances’ (p. 183) [33], while those of the two other analyses with similar findings provide no explanations [5, 25]. What is interesting, is that it was the youngest age-group of women who showed most evidence of greater distress when GRAs and actual roles conflicted. In the introduction to this paper, we noted the suggestion that egalitarian GRAs are taken for granted among younger women [39] and suggested this might mean they are less important for this age-group. However, our analyses suggest the opposite.

We saw some differences in associations between GRAs, roles and psychological distress according to age, but there was almost no evidence of differences between the two dates. This is surprising, given increased egalitarianism, levels of cohabitation and participation of men in chores and of women in the labour market, evident not only in UK society generally over the life-course of the various respondent sub-groups [11, 38], but also when examining our dataset by age and date. One reason might be that although changes in GRAs and roles *did* occur over the 16-year period, they were not large enough to impact on relationships with psychological distress. This is particularly the case for GRAs; for example, among the youngest age groups in our analyses mean traditionalism (on a 1–5 point scale) reduced by around 0.1 points in both men and women between 1991 and 2007. UK data on GRAs are only available from the early 1980s, and an examination of trends from 1980 to 2002 concluded that changes had been ‘surprisingly modest’ (p.167), while acknowledging that there may have been more marked changes before 1980 [50]. It is, therefore, possible that we might have seen more contrast had similar data been collected several decades earlier, at the time of greater political activity around gender equality.

Our study had a number of strengths. Unlike many studies in this area, ours was based on relatively large samples. Our measure of psychological distress, the GHQ-12, is a valid and reliable self-administered screening tool which was designed to detect mental disorders in community samples and has been extensively used in both surveys and clinical settings [45, 46]. Our use of the GHQ-12 as a continuous measure of psychological distress ensured analytic power: if relationships were present, we should have detected them.

There are also a number of limitations, principal among which is that, given the already rather complex nature of the relationships we examined, some of our measures were fairly crude. In particular, we categorised respondents simply as in paid employment or not, rather than separating full- and part-timers. If we had done this, the combined ‘man’ and ‘woman’ employment variable would have been cumbersome. However, accounting for hours worked, particularly among women (since it has been suggested that it is only women’s full-time work which is associated with more equal chore division [1]) might be important. Given evidence of the importance of multiple roles and of work-life balance for well-being [23, 41, 69], analyses examining combinations of ‘couple roles’ might have revealed relationships with psychological distress not evident when examining each independently, as we did here. A second possible limitation is that our measure of chores did not include certain forms of ‘family work’ which some have found to be associated with well-being [22]. However, the chores we included were those identified in other studies as some of the most time-consuming [53] and ‘low-control’ [5], exactly the type of chores which one study found were associated with increased psychological distress [5]. Third, it is possible that had we chosen to focus on satisfaction with the marital/partner relationship (rather than psychological distress) as our dependent variable, we might have found clearer associations between this and our ‘couple role’ measures. Finally, although we controlled for dependent children in the household, analyses based on more detailed categorisations of children were precluded since there were very few/no respondents in the youngest age groups with older children or in the oldest age group with pre-schoolers.

## Conclusion

Consistent with previous studies, gender role attitudes within the British Household Panel Survey around the new millennium were patterned according to gender, age, date and actual household and employment roles, and psychological distress was higher among those with more gender-traditional attitudes and, particularly among men, those not in paid employment. Associations between psychological distress and both marital status and household chore division were only seen in certain sub-groups of women, and it was only among women that we saw the rather weak and inconsistent evidence of lower well-being when GRAs and actual role conflicted. Although this may result from study limitations, it may reflect cultural differences since most previous studies in this area were conducted in the US. Finally, although we observed some different patterns according to age, there were almost none according to date, perhaps because changes in GRAs between 1991 and 2007 were not large enough to impact on relationships with psychological distress.

## Electronic supplementary material

Below is the link to the electronic supplementary material.

Supplementary material 1 (DOC 239 kb)
