# Supplementary material for: Changing gender roles and attitudes and their implications for well-being around the new millennium
Source: Soc Psychiatry Psychiatr Epidemiol. 2013 Aug 2;49(5):791–809. doi: 10.1007/s00127-013-0730-y (PMC4024124; doi:10.1007/s00127-013-0730-y)
Supplement: Supplementary file 1 — Supplementary material 1 (DOC 239 kb) [file 127_2013_730_MOESM1_ESM.doc]

**Supplementary Table 1: Numbers at each date and exclusions to derive final samples for analysis**

|  | **1991**  **(Wave 1)** | **2007**  **(Wave 17)** |
| --- | --- | --- |
| All adults | 10,264 | 14,910 |
| Select respondents who are married/living as a couple | 6,683 | 9,539 |
| Select only those in one-couple households | 6,593 | 9,324 |
| Remove proxy respondents | 6,396 | 8,722 |
| Limit to age 20-64 | 5,452 | 7,023 |
| Remove other adult household members | 5,430 | 6,974 |
| Select only those in heterosexual couples | 5,422 | 6,934 |
| Select respondents with no missing data (final samples for analysis) | 5,302 | 6,621 |

**Supplementary Table 2 – Traditionalism score and ‘couple role variables’, highest qualifications and dependent children: significance of (a) interactions with date among men and women in each age group; and (b) differences between age groups.**

|  |  |  |  |  |  |  |  |  |  |  |  |  |  |
| --- | --- | --- | --- | --- | --- | --- | --- | --- | --- | --- | --- | --- | --- |
|  | **(a) Significance of interactions with date** | | | | | |  | **(b) Significance of differences between regression coefficients in regression models for each age group ^1^** | | | | | |
|  | **AGE 20-34** | | **AGE 35-49** | | **AGE 50-64** | |  | **AGE 20-34**  **vs 35-49** | | **AGE 20-34**  **vs 50-64** | | **AGE 35-59**  **vs 50-64** | |
|  | **Men** | **Women** | **Men** | **Women** | **Men** | **Women** |  | **Men** | **Women** | **Men** | **Women** | **Men** | **Women** |
| **Date (1991)** |  |  |  |  |  |  |  |  |  |  |  |  |  |
| 2007 | *N/A* | *N/A* | *N/A* | *N/A* | *N/A* | *N/A* |  | *0.533* | *0.859* | *0.603* | *0.844* | *0.929* | *0.978* |
|  |  |  |  |  |  |  |  |  |  |  |  |  |  |
| **Marital status (married)** |  |  |  |  |  |  |  |  |  |  |  |  |  |
| Cohabiting | *0.051* | *0.763* | *0.115* | *0.663* | *0.701* | *0.529* |  | *0.176* | *0.180* | *0.429* | *0.155* | *0.818* | *0.615* |
|  |  |  |  |  |  |  |  |  |  |  |  |  |  |
| **Gender balance of chores scale (higher = woman does more)** | *0.065* | *0.016 d* | *0.282* | *0.571* | *0.124* | *0.114* |  | *0.425* | *0.126* | *0.007* | *<0.001* | *0.015* | *0.012* |
|  |  |  |  |  |  |  |  |  |  |  |  |  |  |
| **Couple employment (both work)** |  |  |  |  |  |  |  |  |  |  |  |  |  |
| Only the man works | *0.013 a* | *0.634* | *0.720* | *0.782* | *0.657* | *0.623* |  | *0.963* | *0.347* | *0.003* | *<0.001* | *<0.001* | *<0.001* |
| Only the woman works | *0.186* | *0.172* | *0.748* | *0.689* | *0.832* | *0.110* |  | *0.160* | *0.772* | *0.938* | *0.547* | *0.097* | *0.724* |
| Neither work | *0.182* | *0.057* | *0.131* | *0.578* | *0.292* | *0.168* |  | *0.667* | *0.442* | *0.994* | *<0.001* | *0.588* | *0.002* |
|  |  |  |  |  |  |  |  |  |  |  |  |  |  |
| **Highest qualification (none)** |  |  |  |  |  |  |  |  |  |  |  |  |  |
| Basic secondary school | *0.888* | *0.151* | *0.275* | *0.875* | *0.436* | *0.412* |  | *0.646* | *0.564* | *0.996* | *0.261* | *0.548* | *0.442* |
| University entry level | *0.343* | *0.147* | *0.691* | *0.573* | *0.802* | *0.123* |  | *0.173* | *0.175* | *0.105* | *0.007* | *0.676* | *0.087* |
| University/College | *0.014 b* | *0.001 e* | *0.560* | *0.309* | *0.905* | *0.447* |  | *0.426* | *0.856* | *0.605* | *0.419* | *0.762* | *0.410* |
|  |  |  |  |  |  |  |  |  |  |  |  |  |  |
| **Children in household (none)** |  |  |  |  |  |  |  |  |  |  |  |  |  |
| Any dependent children | *0.027 c* | *0.143* | *0.146* | *0.929* | *0.004 f* | *0.055* |  | *0.017* | *<0.001* | *<0.001* | *<0.001* | *<0.001* | *0.037* |
|  |  |  |  |  |  |  |  |  |  |  |  |  |  |
| *Weighted N* | *1445* | *1884* | *2316* | *2611* | *1706* | *1822* |  | *3761* | *4495* | *3151* | *3666* | *4022* | *4433* |
|  |  |  |  |  |  |  |  |  |  |  |  |  |  |

^1^ = analyses based on unweighted data.

a = significant positive association at both dates, but stronger in 1991; b = significant negative association in 1991, non-significant positive association in 2007; c = significant positive association at both dates, but stronger in 1991; d = significant positive association at both dates, but stronger in 1991; e = significant negative association in 1991, non-significant positive association in 2007; f = non-significant positive association in 1991, significant negative association in 2007.

**Supplementary Table 3 –GHQ likert score and ‘couple role variables’, highest qualifications and dependent children: significance of (a) interactions with date among men and women in each age group; and (b) differences between age groups.**

|  |  | | | | | |  |  | | | | | |
| --- | --- | --- | --- | --- | --- | --- | --- | --- | --- | --- | --- | --- | --- |
|  | **(a) Significance of interactions with date** | | | | | |  | **(b) Significance of differences between regression coefficients in regression models for each age group ^1^** | | | | | |
|  | **AGE 20-34** | | **AGE 35-49** | | **AGE 50-64** | |  | **AGE 20-34**  **vs 35-49** | | **AGE 20-34**  **vs 50-64** | | **AGE 35-59**  **vs 50-64** | |
|  | **Men** | **Women** | **Men** | **Women** | **Men** | **Women** |  | **Men** | **Women** | **Men** | **Women** | **Men** | **Women** |
| **Date (1991)** |  |  |  |  |  |  |  |  |  |  |  |  |  |
| 2007 |  |  |  |  |  |  |  | *0.662* | *0.006* | *0.315* | *<0.001* | *0.540* | *0.080* |
|  |  |  |  |  |  |  |  |  |  |  |  |  |  |
| **Traditionalism score** | *0.713* | *0.971* | *0.344* | *0.642* | *0.823* | *0.359* |  | *0.993* | *0.315* | *0.808* | *0.359* | *0.797* | *0.972* |
|  |  |  |  |  |  |  |  |  |  |  |  |  |  |
| **Marital status (married)** |  |  |  |  |  |  |  |  |  |  |  |  |  |
| Cohabiting | *0.721* | *0.423* | *0.417* | *0.867* | *0.507* | *0.362* |  | *0.710* | *0.067* | *0.962* | *0.860* | *0.841* | *0.274* |
|  |  |  |  |  |  |  |  |  |  |  |  |  |  |
| **Gender balance of chores scale (higher = woman does more)** | *0.059* | *0.764* | *0.423* | *0.962* | *0.021 b* | *0.282* |  | *0.889* | *0.006* | *0.945* | *0.004* | *0.831* | *0.564* |
|  |  |  |  |  |  |  |  |  |  |  |  |  |  |
| **Couple employment (both work)** |  |  |  |  |  |  |  |  |  |  |  |  |  |
| Only the man works | *0.574* | *0.535* | *0.278* | *0.230* | *0.716* | *0.556* |  | *0.115* | *0.747* | *0.096* | *0.646* | *0.901* | *0.456* |
| Only the woman works | *0.018 a* | *0.409* | *0.724* | *0.983* | *0.324* | *0.407* |  | *0.038* | *0.161* | *0.302* | *0.009* | *<0.001* | *0.197* |
| Neither work | *0.900* | *0.218* | *0.081* | *0.379* | *0.840* | *0.896* |  | *0.166* | *0.371* | *0.352* | *0.002* | *0.017* | *<0.001* |
|  |  |  |  |  |  |  |  |  |  |  |  |  |  |
| **Highest qualification (none)** |  |  |  |  |  |  |  |  |  |  |  |  |  |
| Basic secondary school | *0.163* | *0.340* | *0.622* | *0.573* | *0.335* | *0.478* |  | *0.796* | *0.055* | *0.714* | *0.089* | *0.454* | *0.864* |
| University entry level | *0.320* | *0.817* | *0.528* | *0.634* | *0.879* | *0.564* |  | *0.865* | *0.167* | *0.112* | *0.002* | *0.081* | *0.017* |
| University/College | *0.373* | *0.375* | *0.941* | *0.311* | *0.810* | *0.729* |  | *0.543* | *0.048* | *0.125* | *0.145* | *0.009* | *0.610* |
|  |  |  |  |  |  |  |  |  |  |  |  |  |  |
| **Children in household (none)** |  |  |  |  |  |  |  |  |  |  |  |  |  |
| Any dependent children | *0.115* | *0.131* | *0.297* | *0.601* | *0.595* | *0.285* |  | *0.008* | *0.117* | *0.528* | *0.804* | *0.005* | *0.441* |
|  |  |  |  |  |  |  |  |  |  |  |  |  |  |
| *Weighted N* | *1445* | *1884* | *2316* | *2611* | *1706* | *1822* |  | *3761* | *4495* | *3151* | *3666* | *4022* | *4433* |
|  |  |  |  |  |  |  |  |  |  |  |  |  |  |

^1^ = analyses based on unweighted data.

a = significant positive association in 1991, non-significant positive association in 2007; b = non-significant negative association in 1991, significant positive association in 2007.

**Supplementary Table 4: Mutually adjusted relationships with GHQ likert score, including interactions between traditionalism and ‘couple roles’ – men and women in each age group**

|  | **AGE 20-34** | | | |  | **AGE 35-49** | | | |  | **AGE 50-64** | | | |
| --- | --- | --- | --- | --- | --- | --- | --- | --- | --- | --- | --- | --- | --- | --- |
|  | **Men** | | **Women** | |  | **Men** | | **Women** | |  | **Men** | | **Women** | |
|  | Coeff | *Sig* | Coeff | *Sig* |  | Coeff | *Sig* | Coeff | *Sig* |  | Coeff | *Sig* | Coeff | *Sig* |
| **Date (1991)** |  |  |  |  |  |  |  |  |  |  |  |  |  |  |
| 2007 | 0.25 | *0.361* | -0.11 | *0.695* |  | 0.49 | *0.050* | 0.57 | *0.025* |  | 0.76 | *0.006* | 1.17 | *<0.001* |
|  |  |  |  |  |  |  |  |  |  |  |  |  |  |  |
| **Traditionalism** | -0.15 | *0.856* | -0.46 | *0.598* |  | 1.87 | *0.007* | 2.61 | *0.003* |  | 1.25 | *0.145* | 2.45 | *0.027* |
|  |  |  |  |  |  |  |  |  |  |  |  |  |  |  |
| **Marital status (married)** |  |  |  |  |  |  |  |  |  |  |  |  |  |  |
| Cohabiting | -0.50 | *0.710* | -2.95 | *0.012* |  | 0.55 | *0.791* | -1.51 | *0.573* |  | 0.84 | *0.812* | 2.73 | *0.318* |
|  |  |  |  |  |  |  |  |  |  |  |  |  |  |  |
| **Gender balance of chores scale (higher = woman does more)** | -0.25 | *0.458* | -0.37 | *0.293* |  | 0.58 | *0.084* | 0.70 | *0.045* |  | 0.48 | *0.215* | 0.27 | *0.578* |
|  |  |  |  |  |  |  |  |  |  |  |  |  |  |  |
| **Couple employment (both work)** |  |  |  |  |  |  |  |  |  |  |  |  |  |  |
| Only the man works | 1.72 | *0.233* | 3.71 | *0.017* |  | 1.60 | *0.360* | 1.50 | *0.416* |  | -1.19 | *0.471* | 7.83 | *0.002* |
| Only the woman works | 0.23 | *0.941* | -3.60 | *0.331* |  | -0.25 | *0.951* | 2.09 | *0.532* |  | 2.49 | *0.337* | -0.16 | *0.930* |
| Neither work | 1.95 | *0.530* | 4.60 | *0.049* |  | 7.46 | *0.225* | 3.72 | *0.370* |  | 1.98 | *0.442* | 0.90 | *0.642* |
|  |  |  |  |  |  |  |  |  |  |  |  |  |  |  |
| **Highest qualification (none)** |  |  |  |  |  |  |  |  |  |  |  |  |  |  |
| Basic secondary school | -0.22 | *0.599* | -0.69 | *0.091* |  | 0.06 | *0.852* | -0.11 | *0.724* |  | -0.56 | *0.123* | -0.58 | *0.116* |
| University entry level | -0.14 | *0.743* | -0.41 | *0.386* |  | 0.22 | *0.528* | -0.30 | *0.459* |  | -1.05 | *0.004* | 0.25 | *0.651* |
| University/college | 0.08 | *0.861* | -0.49 | *0.336* |  | 0.76 | *0.034* | -0.45 | *0.227* |  | -1.12 | *0.004* | -1.14 | *0.005* |
|  |  |  |  |  |  |  |  |  |  |  |  |  |  |  |
| **Children in household (none)** |  |  |  |  |  |  |  |  |  |  |  |  |  |  |
| Any dependent children | 0.27 | *0.388* | 0.19 | *0.561* |  | -0.35 | *0.220* | 0.18 | *0.550* |  | 1.16 | *0.003* | 0.50 | *0.403* |
|  |  |  |  |  |  |  |  |  |  |  |  |  |  |  |
| **INTERACTIONS WITH TRADITIONALISM** |  |  |  |  |  |  |  |  |  |  |  |  |  |  |
|  |  |  |  |  |  |  |  |  |  |  |  |  |  |  |
| **Traditionalism X marital status (married)** |  |  |  |  |  |  |  |  |  |  |  |  |  |  |
| Cohabiting | 0.21 | *0.663* | 1.18 | *0.010 a* |  | -0.22 | *0.758* | 0.89 | *0.378* |  | -0.27 | *0.828* | -0.94 | *0.327* |
|  |  |  |  |  |  |  |  |  |  |  |  |  |  |  |
| **Traditionalism X chores** | 0.08 | *0.549* | 0.16 | *0.200* |  | -0.18 | *0.111* | -0.32 | *0.012 a* |  | -0.11 | *0.379* | -0.18 | *0.274* |
|  |  |  |  |  |  |  |  |  |  |  |  |  |  |  |
| **Traditionalism X couple employment (both work)** |  |  |  | *a* |  |  |  |  |  |  |  |  |  | *a* |
| Only the man works | -0.39 | *0.408* | -1.10 | *0.033* |  | -0.55 | *0.319* | -0.35 | *0.556* |  | 0.24 | *0.649* | -2.47 | *0.002* |
| Only the woman works | 1.13 | *0.339* | 2.40 | *0.079* |  | 1.76 | *0.190* | -0.47 | *0.684* |  | -0.33 | *0.710* | -0.06 | *0.933* |
| Neither work | 0.28 | *0.796* | -0.83 | *0.285* |  | -0.78 | *0.683* | -0.21 | *0.876* |  | -0.05 | *0.953* | -0.21 | *0.757* |
|  |  |  |  |  |  |  |  |  |  |  |  |  |  |  |

a = addition of this interaction term resulted in improvement of model fit p<0.05
